# Deletion of *afpab1* Causes Increased Sensitivity to Oxidative Stress and Hypovirulence in *Aspergillus fumigatus*

**DOI:** 10.3390/ijms17111811

**Published:** 2016-10-29

**Authors:** Dongyang Wang, Shunan Wang, Dan He, Song Gao, Baiji Xue, Li Wang

**Affiliations:** Department of Pathogenobiology, Jilin University Mycology Research Center, Key Laboratory of Pathobiology, Ministry of Education, College of Basic Medical Sciences, Jilin University, Changchun 130021, China; wdyang123456@sina.com (D.W.); wsnzhenjun@sina.com (S.W.); hedanzhenjun@sina.com (D.H.) gaosongzhenjun@sina.com (S.G.); xuebaijizhenjun@sina.com (B.X.)

**Keywords:** *Aspergillus fumigatus*, *Agrobacterium tumefaciens*, *afpab1*, oxidative stress, survival factor

## Abstract

*Aspergillus fumigatus* AFPAB1 is the ortholog of the *Aspergillus oryzae* cytoplasmic messenger ribonucleoprotein granules AOPAB1 that function to depress the initiation of translation during stress. *A. fumigatus* can regulate its cellular physiology in response to environmental stresses, but there has been no research on Pab1 in *A. fumigatus*. The associated gene *afpab1* was replaced with a hygromycin-selectable marker to generate the strain *Δafpab1*. Phenotypic analysis showed that the *Δafpab1* grew more weakly than the wild-type strain. Also the germination rate of *Δafpab1* was decreased when exposed to oxidative stress. The morphology of *Δafpab1* spores also showed great changes. The killing rate of *Δafpab1* by RAW264.7 murine macrophage cells was increased, and the reactive oxygen species (ROS) scavenging ability of *Δafpab1* was decreased. Pathogenicity testing showed that the deletion strain had decreased virulence. Therefore, we conclude that *afpab1* activity is correlated with susceptibility to oxidative stress, and deletion of *afpab1* from *A. fumigatus* possibly leads to observed hypovirulence in an immunosuppressed mouse model.

## 1. Introduction

*Aspergillus fumigatus* (*A. fumigatus*) is a widespread saprophytic fungus that produces large numbers of asexual spores. It is an opportunistic pathogen with a worldwide distribution. Cases of *A. fumigatus* infection have increased markedly over recent decades. It has become the most important aerial fungal pathogen of humans, causing disorders dependent on the host immune response [1]. In healthy hosts, spores are usually harmless because they are eliminated by the human immune system [2]. However, in immunocompromised individuals, *A. fumigatus* conidia can cause aspergillosis. Research into *A. fumigatus* virulence has therefore become increasingly important.

The external environment of *A. fumigatus* in infection conditions is different to that in its normal growth niche [3]. The ability to sense external stress and respond appropriately is vital for the survival of fungal cells in stress conditions, and helps *A. fumigatus* to establish successful invasive aspergillosis. Global translational inhibition is one of the main mechanisms by which eukaryotic cells resist external stress [4]. Eukaryotic cells shut down the transcription process and accumulate stalled translation components in cytoplasmic messenger ribonucleoprotein granules, which typically occur when cells are exposed to stress and form in response to the inhibition of translation initiation [5]. Non-translated mRNA and related proteins are collected from polysomes into cytoplasmic messenger ribonucleoprotein granules when cells encounter stress conditions [6,7]. It has been speculated that cytoplasmic messenger ribonucleoprotein granules function as transcription repositories that regulate the translatability and stability of mRNAs in stress conditions [8,9].

Pab1 is a cytoplasmic messenger ribonucleoprotein granule that has been widely studied in yeast cells and is conserved in eukaryotes [10]. A recent study has shown that Pab1 exists in the filamentous fungus *Aspergillus oryzae* [5]. The function of Pab1 in *A. oryzae* is to resist external stresses including oxidative stress, temperature stress and endoplasmic reticulum (ER) stress (protein folding in the ER is impaired resulting in the accumulation of misfolded proteins) [11]. However, there has been no research on Pab1 in *A. fumigatus*, and the ability to cope with external stress is one of the most important survival factors of *A. fumigatus*. Therefore, in the present study we knocked out the *A. fumigatus* gene *afpab1*, a homolog of *A. oryzae aopab1*, to investigate its role in the response of *A. fumigatus* to stress, and to determine whether it has any effect on pathogenicity.

## 2. Results

### 2.1. Isolation and Sequence Analysis of afpab1 Homolog from A. fumigatus

**Figure 1:**
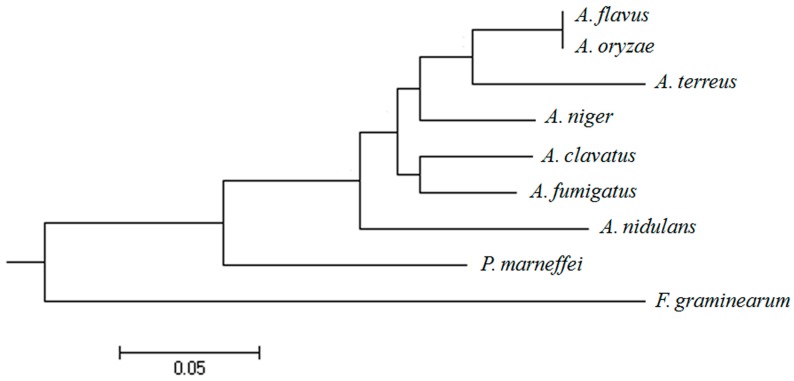
In silico analysis of the *afpab1* in *A. fumigatus*. Protein alignment was performed with ClustalX 2 and the phylogenetic tree was created via MEGA 5.2 to examine *afpab1* homologs from different fungi (*Aspergillus fumigatus*, AFUA_1G04190; *Aspergillus oryzae*, AO090003000927; *Aspergillus clavatus*, ACLA_030250; *Aspergillus flavus*, AFLA_028910A; *Aspergillus niger*, An01c0080; *Aspergillus terreus*, ATEG_03789; *Aspergillus nidulans*, AN4000.2; *Penicillium marneffei*, PMAA_054700; *Fusarium graminearum*, FGRRES_08421M).

A BLAST search with *A. oryzae aopab1* as the query identified a predicted cytoplasmic messenger ribonucleoprotein granule protein *afpab1* in the *A. fumigatus* genome. The coding sequence was identified by cloning and sequencing cDNAs using RT-PCR. The putative *afpab1* gene was located on chromosome 1, and consisted of 2396 bp (with 134 bp in two introns and 2262 bp in three exons), corresponding to 753 amino acid residues. The phylogenetic relationships among *pab1* genes from different fungi were analyzed and the results suggest that the *pab1* gene is present in a variety of fungi of different species (Figure 1). These results demonstrate that the *afpab1* gene had significant homology with that of *A. clavatus*, and minimal similarity to the gene from *Fusarium graminearum*.

### 2.2. Deletion and Complementation of afpab1 in A. fumigatus

**Figure 2:**
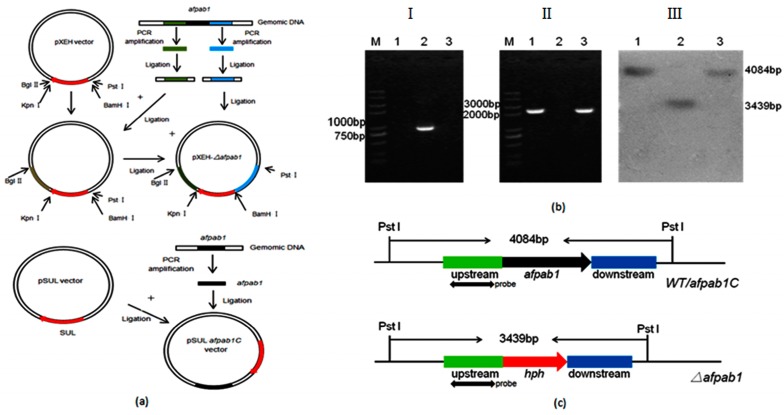
Deletion and reconstitution of *afpab1* of *A. fumigatus*. (**a**) The constructed schematic of *Δafpab1* and *afpab1C* in *A. fumigatus*; (**b**) Molecular analysis of WT, *Δafpab1* and *afpab1C*. *Hph* gene can be amplified from *Δafpab1* (I) but cannot be amplified from WT or *afpab1C*, and *afpba1* gene can be amplified from WT and *afpab1C* (II) which cannot be amplified from *Δafpab1*. Genomic DNA digested with Pst I was probed with an upstream region of *afpab1* to detect the *Δafpab1*, WT, and *afpab1C* (III); (**c**) Southern hybridization schematic diagram. M: DNA molecular size marker Trans 2K plus II; 1: WT; 2: *Δafpab1*; 3: *afpab1C*. (Green: upstream fragment of *afpab1* gene, Red: *hph* gene, Blue: downstream fragment of *afpab1* gene).

To determine the function of Afpab1 from *A. fumigatus*, a *Δafpab1* deletion strain was constructed by replacing the entire gene with *hph* (Figure 2a). The *hph* gene could be amplified from *Δafpab1* using the primers Hphf/Hphr, but could not be amplified from the WT. Conversely, the *afpab1* gene could be amplified from the WT, but not from *Δafpab1*, using specific primers (Figure 2b). A schematic and the results of Southern hybridization are shown in Figure 2b,c. The *Δafpab1* strain showed a 3.4 kb hybridization band that was not present in WT and complementation strain.

### 2.3. Growth and Phenotype of the Δafpab1 Strain

To determine whether disruption of *afpab1* contributed to any growth defects, the growth of *Δafpab1* was examined. There were no significant variations in the growth of the deletion strain. In addition, there was no difference in colony morphology by direct observation or under light microscopy (Figures S1–S3).

### 2.4. Δafpab1 Shows Increased Sensitivity to Oxidative Stress

**Figure 3:**
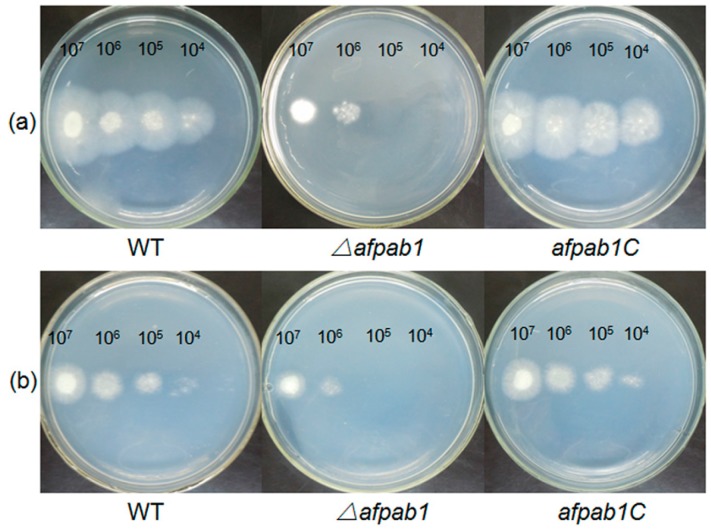
Plates assay. The *A. fumigatus* WT, *Δafpab1* and *afpab1C* strains were dot inoculated onto PDA at a gradient of 10-fold dilution for 36–48 h at 28 °C. (**a**) Medium containing 1.5 mM H_2_O_2_; (**b**) Medium containing 10 µM menadione.

**Figure 4:**
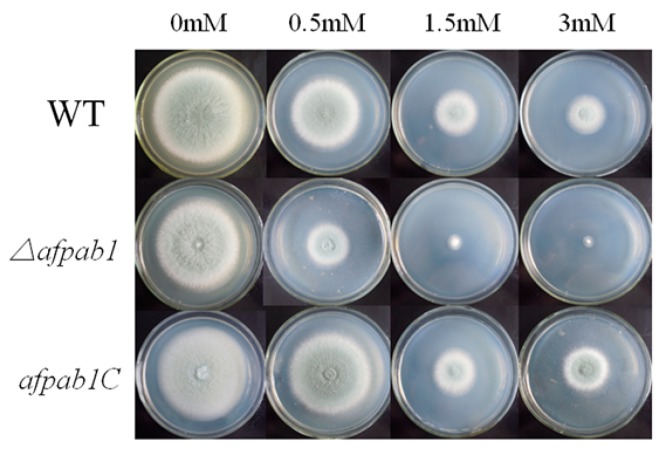
Sensitivity of WT, *Δafpab1* and *afpab1C* to H_2_O_2_ stress conditions. Five microliters of conidial saline suspensions (1 × 10^6^ cells/mL) were inoculated onto PDA. Cultures were incubated at 28 °C for 4 days.

**Figure 5:**
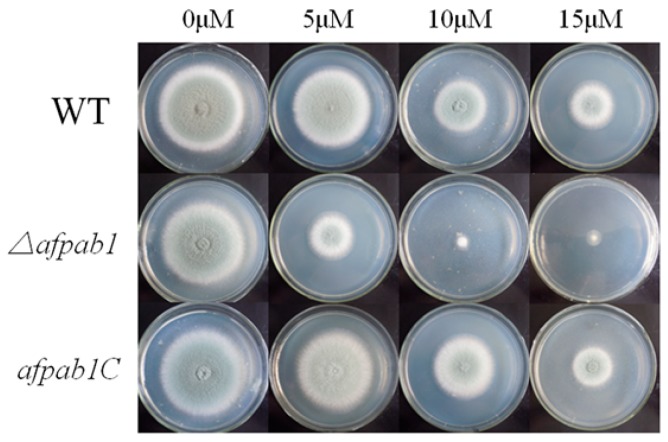
Sensitivity of WT, *Δafpab1* and *afpab1C* to menadione stress conditions. Five microliters of conidial saline suspensions (1 × 10^6^ cells/mL) were inoculated onto PDA. Cultures were incubated at 28 °C for 4 days.

**Figure 6:**
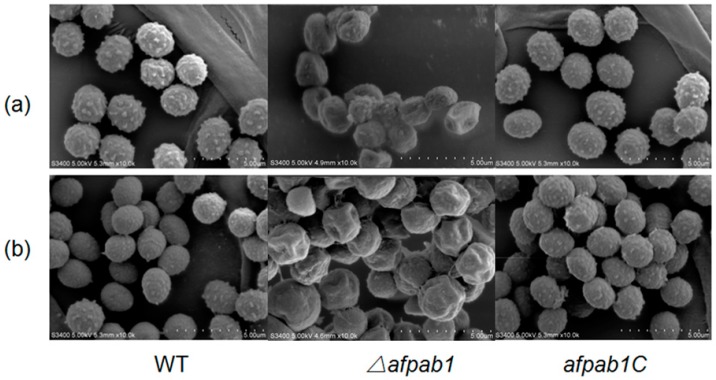
The coverslips covered on PDA cubes with spores were transferred and prefixed with 2% glutaraldehyde at room temperature for 12 h and then metalized with gold and investigated by scanning electron microscope. (**a**) Medium containing 1.5 mM H_2_O_2_; (**b**) Medium containing 10 µM menadione.

No differences were detected between *Δafpab1* and the WT under osmotic, ER or temperature stress conditions (Shown in Figure S4). Interestingly, the *Δafpab1* showed greater sensitivity to H_2_O_2_ and menadione than the WT and *afpab1C* (Figure 3, Figure 4 and Figure 5). We also found under scanning electron microscope (SEM) that the spore surfaces were smoother (fewer ornaments), depressed, deformed, and irregularly shaped compared with the WT and *afpab1C* (Figure 6).

**Figure 7:**
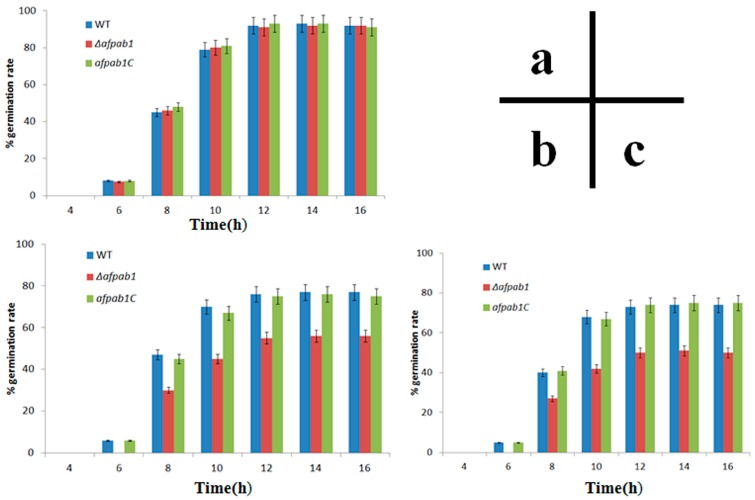
Germination rates of *afpab1* deletion strain compared with WT and *afpab1C* under control, 3 mM H_2_O_2_ or 15 µM menadione. Data shown (means ± SD) are from three independent experiments for each strain. (**a**) Control; (**b**) H_2_O_2_; (**c**) Menadione.

As shown in Figure 7, *Δafpab1* displayed a significantly reduced rate of germination under oxidative stress. After 12 h incubation, there was no difference in the control group. The germination rates of WT, *Δafpab1*, and *afpab1C* were 77%, 56%, and 76% when medium contained 3 mM H_2_O_2_. Under 15 µM menadione, the germination rates of these strains were 74%, 50%, and 75%, respectively.

### 2.5. ROS Production of Δafpab1 Is Increased When Exposed to H_2_O_2_

**Figure 8:**
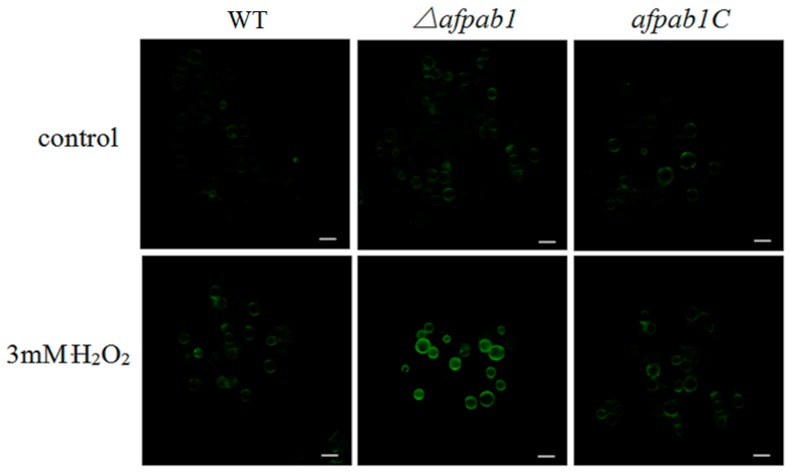
DCFH-DA staining was used to determine the ROS levels in the protoplasts treated with 3 mM H_2_O_2_ for 1 h. The conidia were observed using fluorescence microscopy to determine the ROS levels; Control group without H_2_O_2_. Bar, 10 µm.

**Figure 9:**
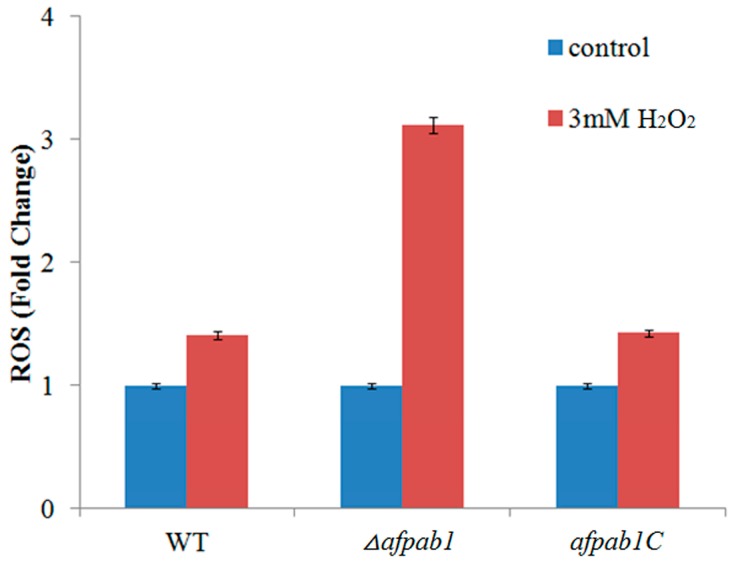
The ROS fluorescence intensity was analyzed by software Image-Pro Plus 6.0. The fluorescence intensity was equal in the control group, but the fluorescence intensity of *Δafpab1* increased nearly two fold to that of WT and *afpab1C* after the treatment of 3 mM H_2_O_2_. Data were shown as the mean ± SD, *p* < 0.05.

ROS can convert DCFH into 2,7-dichlorofluorescein (DCF), and DCF is fluorescent. However, DCFH-DA are often used in animal cells or tissues, but are rarely used in fungi cells [12]. Animal cells do not have cell walls, but fungal cells do. We therefore prepared protoplasts to measure the fluorescence of each strain. Enzymatic hydrolysis was carried out under optimum conditions with 5 mg/mL lallzyme, 1 mg/mL cellulase, and 1 mg/mL helicase at 28 °C for 150 min. Green fluorescence was observed with confocal laser micro-scopy. The *Δafpab1* deletion strain had stronger green fluorescence than the WT and *afpab1C* when treated with 3 mM H_2_O_2_ for 1 h (Figure 8). Analysis with Image-Pro Plus 6.0 showed that the *Δafpab1* fluorescence intensity increased nearly twice as much as that of WT and *afpab1C* under treatment with 3 mM H_2_O_2_ (Figure 9).

### 2.6. Killing Rate for Δafpab1 Is Increased in RAW264.7 Cells

**Figure 10:**
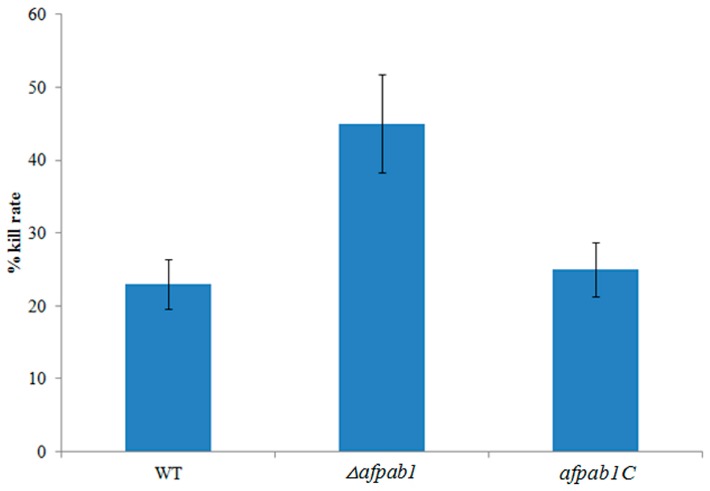
Percentage of killing by macrophage cells of conidia obtained from WT, *Δafpab1*, and *afpab1C*. The killing rate for each strain was determined from three independent experiments. Data were shown as the mean ± SD, *p* < 0.05.

Immune cells secrete ROS to kill conidia. A macrophage–conidia co-culturing method was used to examine the ability of *A. fumigatus* to resist immune cell oxidative stress. As shown in Figure 10, the killing rate for *Δafpab1* was 45%. By contrast, only 23% and 25% of conidia from WT and *afpab1C* were killed.

### 2.7. Murine Model of Invasive Pulmonary Aspergillosis

**Figure 11:**
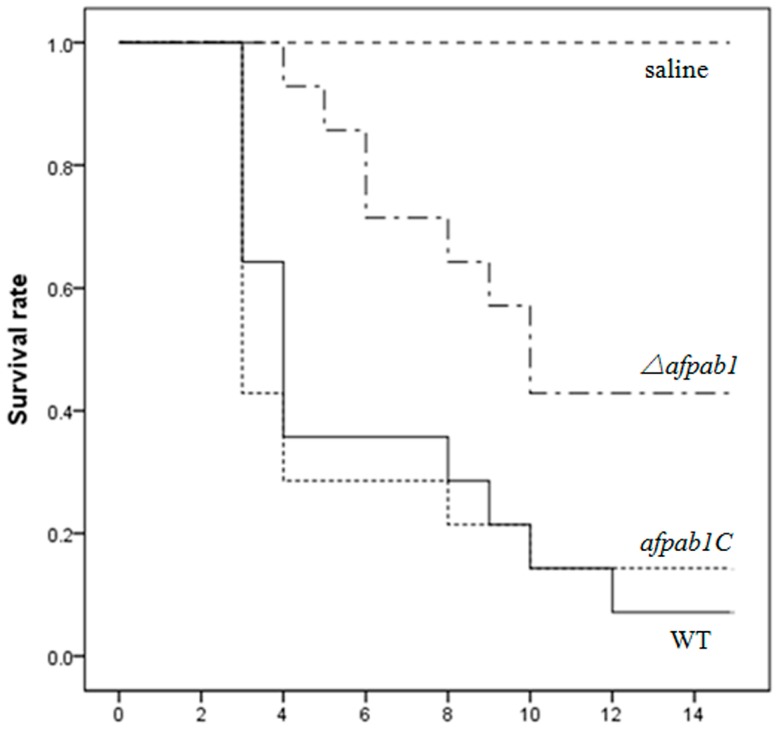
Kaplan-Meier analysis showed that survival rate of mice infected with *Δafpab1* was significantly higher than the WT and *afpab1C* (*p* < 0.05).

**Figure 12:**
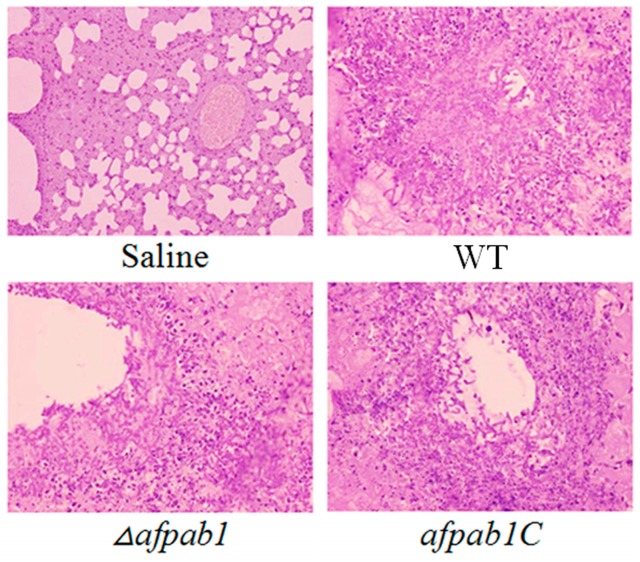
Lung sections from the dead mice were Periodic acid-Schiff stained and photographed (100×). The result showed that the mice died of invasive aspergillosis.

The survival rate of mice infected with physiological saline was 100% at the end of the assay, whereas 10% of mice infected with the WT and 15% of mice infected with the complementation strain survived to the 14th day. The survival rate of mice infected with *Δafpab1* was 43%, showing a significant difference (*p*-value = 0.008; Figure 11). All infected animals displayed progressive and severe signs of invasive disease, including ruffled fur, hunched posture, and an increased respiratory rate [13]. The mice that died from each group were dissected to separate the lungs for periodic acid-Schiff staining. As shown in Figure 12, there was extensive destruction of the bronchus wall and alveoli by mycelium invasion, meaning that the mice died of aspergillosis. Mice infected with the *Δafpab1* strain showed fewer hyphae in lung sections.

## 3. Discussion

*Aspergillus fumigatus* is a ubiquitous opportunistic pathogen. In recent decades, with the use of immunosuppressants after organ transplantation, cases of *A. fumigatus* infection causing mycosis or invasive aspergillosis in immunocompromised patients have increased [14]. The mortality rate of aspergillosis caused by *A. fumigatus* has increased dramatically [15]. Some survival factors of *A. fumigatus* have been identified, such as coping with oxidative stress and iron acquisition [3].

Coping with oxidative stress has great significance in many fungi. Dong’s research [16] suggests that in *Candida albicans CCZ1* functions in oxidative stress, and deletion of *CCZ1* leads to an increased sensitivity to macrophages. It is well accepted that the ability to resist oxidative stress is necessary for *A. fumigatus* to survive and establish a successful infection. Studies have identified some genes, such as *sod1* and *afyap1*, to be associated with oxidative stress resistance and ROS protection [17]. In Lessing’s research [18], deletion of the Afyap1 gene led to drastically increased sensitivity to H_2_O_2_ and menadione. Lambou found that the triple *sod1*/*sod2*/*sod3* (fungal superoxide dismutases) deletion strain was characterized by a delay in conidial germination, reduced conidial survival during storage over time, the highest sensitivity to menadione, and an increased sensitivity to alveolar macrophages of immunocompetent mice.

Pab1 is a cytoplasmic messenger ribonucleoprotein granule, and it has been confirmed that Pab1 is related to the oxidative stress response in *A. oryzae* [5]. However, prior to the present study, there had been no research on Pab1 in the opportunistic pathogenic fungus *A. fumigatus*. Thus, to study the function of *afpab1* in *A. fumigatus*, we constructed a *Δafpab1* deletion strain by targeted gene disruption, along with a complemented deletion strain by introduction of a complementation plasmid.

Compared with the WT strain, the *Δafpab1* deletion strain showed no difference in growth rate or sensitivity to heat stress, osmotic pressure stress, and ER stress, but increased sensitivity to oxidative stress. In this study, we observed that *Δafpab1* was more sensitive to H_2_O_2_ and menadione than the wild-type strain. The *Δafpab1* conidial morphology changed greatly under the SEM when it was treated with H_2_O_2_ and menadione. Also, we also found the germination rate of *Δafpab1* was decreased compared with WT and *afpab1C* under oxidative stress.

ROS derived from immune cells is the main mechanism for the elimination of conidia in humans. Previous research has revealed that ROS are toxic to fungal invaders [19,20]. Hence, molecules associated with ROS resistance can be considered as survival factors. Studies have reported that *A. fumigatus* cells can be killed and phagocytosed by immune cells, which can produce strong reactive oxygen agents [21]. Eukaryotic cells can develop oxidative defense systems and synthesize a variety of molecules, including catalase, superoxide dismutases, and glutathione peroxidase, to protect cells from damage by oxidative agents [17,22]. In this research, a macrophage-conidia co-culturing method was used to examine the ability of *A. fumigatus* to resist immune cell oxidative stress. We found that the killing rate of *Δafpab1* by macrophages was increased compared with WT and *afpab1C*, indicating that the ability to resist ROS damage is decreased in *Δafpab1*.

To evaluate the ability of *Δafpab1* to resist ROS, we used DCFH-DA staining methods. The results showed that the protoplasts of each strain had less fluorescence when H_2_O_2_ was absent. When the protoplasts were treated with H_2_O_2_, *Δafpab1* showed marked ROS fluorescence compared with the WT and *afpab1C*. We speculate that this was owing to the deletion of *afpab1*, the *Δafpab1* cells having decreased ability to scavenge ROS, meaning that ROS metabolism is affected in *Δafpab1*.

In this study, we found that deletion of *afpab1* causes increased sensitivity to oxidative stress in *A. fumigatus*. However, the mechanism of Pab1 in oxidative stress resistance is still unknown. There has been little research related to Pab1. Roque’s [23] study found that eukaryotic release factor 3 (eRF3) is implicated in translation termination and also interacts with poly-A binding protein (PABP, the yeast homolog of Pab1). The results show that in *Saccharomyces cerevisiae* the role of the Pab1 C-terminal domain in mRNA stability is independent of eRF3 and the association of these two factors negatively regulates translation termination.

Some genes with ROS protection functions have been found, and their deletion leads to *A. fumigatus* hypovirulence in animal models [24]. We speculate that Pab1 might influence the post-transcriptional translation of these genes in oxidative stress conditions, and that lack of *afpab1* makes *A. fumigatus* more sensitive to oxidative stress. In this study, we evaluated the pathogenicity of *Δafpab1* in an immunosuppressed mouse model, and found that it is decreased compared with the WT and *afpab1C* because of increased sensitivity to oxidative stress and decreased ROS protection ability. We conclude that *afpab1* is related to the oxidative stress response, and that loss of this gene causes *A. fumigatus* hypovirulence in the immunosuppressed mouse model because the protection of cells from oxidative stress damage is one of the most important virulence factors in *A. fumigatus*.

## 4. Experimental Section

### 4.1. Strains, Media, and Growth Conditions

The wild-type (WT) strain *A. fumigatus* AF293 (purchased from ATCC, Manassas, VA, USA) was used for all in vitro and animal model experiments, the bacteria strain (*Escherichia coli* DH5α and *Agrobacterium tumefaciens* AGL-1) were preserved at the Jilin University Mycology Research Center. Strains were grown in potato dextrose agar (PDA) medium (potato 200 g, glucose 20 g, agar 20 g, H_2_O 1000 mL). *Escherichia coli* and *Agrobacterium tumefaciens* strains were used for the cloning process and grown in Luria–Bertani broth (tryptone 10 g, yeast extract 5 g, NaCl 10 g, H_2_O 1000 mL) at 37 °C or 28 °C. Media were purchased from Difco (Franklin Lakes, NJ, USA).

### 4.2. Molecular Cloning of A. fumigatus afpab1

A homolog of *A. oryzae aopab1* (NCBI Gene ID: 5992028) was identified in *A. fumigatus* by performing a Basic Local Alignment Search Tool (BLAST) search at the NCBI website (http://blast.ncbi.nlm.nih.gov/). Total RNA was extracted from *A. fumigatus* wild type strain, according to the Trizol (Invitrogen, Shenyang, Liaoning, China) method. cDNA was generated using a Takara (Dalian, China) reverse transcription kit according to the manufacturer’s instructions. Using primers PabIf and PabIr to amplify the entire coding region of *afpab1*. The Gene ID of the *A. fumigatus afpab1* gene is 3507350.

### 4.3. Construction of the Δafpab1 Deletion Strain and Complementation Strain

A gene knockout vector was constructed by cloning a 949 bp fragment upstream (primers PabUf and PabUr) and a 964 bp fragment downstream (primers PabDfi and PabDri) of the *A. fumigatus afpab1* gene into the plasmid pXEH (containing the hygromycin resistance gene) to generate the knockout plasmid K. Plasmid K was transformed into competent *Ag. tumefaciens AGL-1* using the freeze–thaw method [25]. The resulting strain was designated *At-pab1*. A *Δafpab1* deletion strain was constructed by *Agrobacterium tumefaciens*-mediated transformation as described previously [26,27].

To ensure that the deletion strain phenotype was attributable to the specific deletion, the *afpab1* gene was provided in trans to generate a complementation strain, *afpab1C*. The complementation vector was constructed by cloning a 4193 bp DNA fragment (primers PabCF and PabCB), from 1200 bp upstream to 600 bp downstream of the *afpab1* coding region, into the plasmid pSUL (sulfonylurea resistance), generating plasmid C. Plasmid C was transformed into *Ag. tumefaciens AGL-1*, and the resulting *Ag. tumefaciens* strain was used to transform the *Δafpab1* strain. The complemented strain was confirmed by screening on medium containing sulfonylurea (DingGuo, Changchun, China).

The deletion strain were screened by PCR, using primers to amplify the *afpab1* (Pabr and Pabf) and *hph* (Hphr and Hphf) PCR products from their DNA. The constructed strains were confirmed by Southern blot analysis with Pst I-digested genomic DNA. A 960 bp upstream fragment was used as a probe for hybridization (PabSr and PabSf).

Primers were synthesized by Invitrogen (Shanghai, China) and showed in Table 1.

### 4.4. Growth Analysis and Morphological Observation

Conidia were harvested from each strain grown on potato dextrose agar (PDA) plates and resuspended in 0.01% Tween 80 saline to a final concentration of 1 × 10^6^ spores/mL. To measure growth rates, 5 µL spore suspension was spotted onto the center of a plate of PDA and incubated at 28 °C for 4 days; then the diameter of colonies was observed at various time points. In order to compare the microscopic morphology among the stains, morphology was observed under microscope at 400× magnification.

### 4.5. Plate Assay

To examine the effects of *afpab1* deletion under various stresses encountered during human infection, conidia were collected from the *A. fumigatus* WT, *Δafpab1*, and *afpab1C* strains and suspended in saline. The conidial density was adjusted to a final concentration of 1 × 10^7^ cells/mL. Ten-fold serial dilution of each suspension was carried out to achieve concentrations of 10^6^, 10^5^, and 10^4^ cells/mL for each strain. The spore suspensions (5 μL) were subjected to various stresses. For temperature stress, cells were spotted on PDA medium and incubated at 42, or 48 °C for 36–48 h. Many reagents can be used to perform stress assay. In this study, we used dithiothreitol for ER stress, sorbitol for osmotic stress, H_2_O_2_ and menadione for oxidative stress. For ER, osmotic, and oxidative stresses, conidial suspensions were spotted on culture media supplemented with various substances: Dithiothreitol (5, 10, and 15 mM) (Sigma, St. Louis, MO, USA), sorbitol (0.5, 1, and 1.5 M) (Jin Tai, Changchun, China), H_2_O_2_ (0.5, 1.5, and 3 mM) (Jin Tai), or menadione (5, 10, and 15 µM) (Jin Tai), for 36–48 h at 28 °C.

Drop plates were prepared by spotting 5 µL conidial saline suspensions (1 × 10^6^ cells/mL) onto PDA medium under the conditions described above. The plates were incubated at 28 °C for 4 days and photographed.

### 4.6. Scanning Electron Microscopy

Conidia of the WT, *Δafpab1*, and *afpab1C* strains were inoculated onto 1 cm^3^ PDA blocks (containing 3 mM H_2_O_2_ and 15 µM menadione) covered with coverslips (treated with polylysine), and incubated for 3 days at 28 °C. The coverslips were prefixed with 2% glutaraldehyde (Jin Tai) at 4 °C for 12 h and then metalized with gold and scanned by S-3400N electron microscopy (conditions: Vacc = 5 kV, Work Distance = 4.7 mm), Hitachi (Tokyo, Japan). Each group was repeated in triplicate.

### 4.7. Rates of Germination under Oxidative Stress

Germination rates were assessed according to a previously described method [28] with modifications. Each strain of conidia at a concentration of 10^6^ conidia/mL were incubated in potato dextrose broth in three groups (control, containing 3 mM H_2_O_2_ or 15 µM menadione) at 28 °C, 180 rpm. Rates of germination were detected at 4, 6, 8, 10, 12, 14, and 16 h by microscopic examination. Spores were deemed germinated when the germ tube was equal in size to the conidium. The number of germ-tube-forming spores per random 100 conidia was counted. The experiments were repeated three times.

### 4.8. Protoplast Preparation

The conidial suspension density of each strain was adjusted to a final concentration of 1 × 10^7^ cells/mL in saline with 0.4% Tween 80, and added to 10 mL of potato dextrose broth culture. The cultures were centrifuged at 2400× *g* for 15 min when the rate of germination reached 80%. The remaining products were treated with 4 mL 1.2 M sorbitol-10 mM potassium phosphate (pH = 5.8), containing 5 mg/mL lywallzyme (GIMCC, Guangzhou, China), 1 mg/mL cellulase (Sangon Biotech, Shanghai, China), and 1 mg/mL helicase (Sangon Biotech, Shanghai, China). The mixtures were incubated on a shaking table at 28 °C, 150 rpm. The incubation was monitored by microscopy, and when a large proportion of protoplasts was obtained, the mixture was centrifuged at 2000× *g* for 20 min. The deposits were resuspended in 0.6 M KCl (Jin Tai, Changchun, China) and centrifuged at 2000× *g* for 20 min. Then the supernatants were discarded, and the deposits were resuspended in 10 mM Tris-HCl (pH = 7.5) and 1 M sorbitol.

### 4.9. 2,7-Dichlorofluorescin Diacetate Staining

The protoplast does not have cell walls. For this assay, we followed a previously published method, which used protoplasts to increase reagents to cross the cell membrane. Briefly, reactive oxygen species (ROS) can convert 2,7-dichlorofluorescin diacetate (DCFH-DA) (Sigma, St. Louis, MO, USA) into 2,7-dichlorofluorescein (DCF), which is fluorescent. The protoplast samples from each strain were mixed with 0.1% DCFH-DA, incubated at 37 °C for 30 min, and centrifuged at 4000× *g* for 20 min. The supernatant was discarded and resuspended in regeneration buffer, and then 3 mM H_2_O_2_ was added into the protoplast solution of each strain for 1 h. The ROS levels of the conidia were visualized with an Olympus (Beijing, China) FV1000 confocal laser microscope (Excitation Wavelength of 488 nm and Emission Wavelength of 525 nm). The fluorescence intensity was analyzed using the software Image-Pro Plus 6.0.

### 4.10. Macrophage Assay for Fungal Spores

Macrophage assays were performed as described previously [29]. Briefly, after allowing 1 × 10^4^ of murine RAW264.7 macrophage to adhere for 2 h, 500 µL of WT, *Δafpab1*, or *afpab1C* spores suspension was added to each well. The spore suspension was 1 × 10^5^ conidia/mL in RPMI. The spore suspension density was chosen in order to present 5 spores per macrophage. The macrophages were incubated for 1 h at 37 °C in 5% CO_2_ to perform the phagocytosis stage of the assay. Afterwards, medium was collected from each well in a 5 mL Eppendorf tube, then the well was washed two times with phosphate buffer saline (PBS), which was added to the collected medium. For the spore survival stage of the assay, the macrophages were incubated for 5 h in 500 µL of medium containing 10% fetal bovine serum. Finally, the cell media was collected in a 5 mL Eppendorf tube and the macrophages were lysed with 500 µL of pancreatin lysis buffer (DingGuo, Changchun, China) for 10 min. Lysis buffer was collected into the respective tubes and the wells were washed once with 500 μL PBS, which was similarly collected. The collected suspensions at the different time points (1 and 5 h) were diluted at a 1:20 ratio and 20 μL of the solution was plated on two plates of PDA and grown at 28 °C for 48 h in order to quantify the number of colonies originated from live single spores.

### 4.11. Pathogenicity Assay

A virulence assay was performed as described previously [30], with some modifications. The WT, *Δafpab1*, and *afpab1C* strains were used to infect white male BALB/c mice (Vital River, Beijing, China; 20–22 g). The mice were housed at the Institutional Animal Center, Jilin University, China. Each strain of conidia was suspended in saline to give an inoculum of 3 × 10^5^ cells/g of mouse body weight in 30 µL volume. Immunosuppression was induced in the mice by subcutaneous injection of hydrocortisone acetate (40 mg/kg of body weight) on Day 1 and intraperitoneal injection of cyclophosphamide (150 mg/kg of body weight) on Day 3 and Day 1. Immunosuppression was prolonged by cyclophosphamide injections (150 mg/kg) on days 3, 6, and 9. Mice were kept in sterile cages with filter tops and received sterile food and bedding. Tetracycline (1 mg/mL) and uridine (100 mM) were added to the drinking water, which was changed twice daily. On day 0, mice were anesthetized by inhalation of diethyl ether and infected intranasally with spores in 30 µL volume containing 6 × 10^6^ conidia. Four groups (WT, *Δafpab1*, *afpab1C*, and control) each containing 15 mice were inoculated and monitored twice daily for 14 days to assess mortality. The survival rates were analyzed using the Kaplan–Meier estimator, using SPSS version 13. *p*-values less than 0.05 were considered statistically significant. The lungs from each mouse including those that died before 14 days were removed and stained by the periodic acid-Schiff method, according to the standard procedure [31].

All animal procedures were conducted in compliance with the guidelines of the China Association of Laboratory Animal Care. All animal experiments were approved by the Animal Care and Use Committee at Jilin University (30 September 2015).

### 4.12. Statistics

The data were displayed as means ± SD. The figures represented three independent experiments. Analysis of variance was used to estimate the differences among the groups. *p* < 0.05 was considered statistically significant.

## 5. Conclusions

Our results show that deletion of *afpab1* results in increased sensitivity to oxidative stress. We have also demonstrated that deletion of *afpab1* results in hypovirulence of *A. fumigatus*. This work should facilitate a deeper understanding of the oxidative stress response of *A. fumigatus* and provide a basis for future studies into this unexplored area of its biology.

## Figures and Tables

**Table 1:** Primers used in this study.

Primer Name	Nucleotide Sequence(5′ to 3′)
PabIf	5′-ATGTCTGCCGAAGTCTCTAC-3′
PabIr	5′-CGACTTGTTCTCTTCCGTAG-3′
PabDfi	5′-CTGCAGCCAGTGAAGACCAGGATC-3′
PabDri	5′-GGATCCGACGGACGAGGTGAAGTG-3′
PabUf	5′-GGTACCTCTTCCTATCCCGATTACAT-3′
PabUr	5′-AGATCTATCAACCGTCCCTCACTC-3′
Pabf	5′-GGACCACGAATACCCTGAC-3′
Pabr	5′-CTGCTTCCCTCTATGTCGG-3′
Hphf	5′-CGCCCAAGCTGCATCATCGAA-3′
Hphr	5′-CGACAGCGTCTCCGACCTGA-3′
PabCF	5′-ACTAGTGCGGCGAGGATAGATTAC-3′
PabCB	5′-TCTACAGCGGCGAGGATAGATTAC-3′
PabSr	5′-TCTTCCTATCCCGATTACAT-3′
PabSf	5′-ATCAACCGTCCCTCACTC-3′

## Supplementary Materials

Supplementary materials can be found at www.mdpi.com/1422-0067/17/11/1811/s1.
