# Supplementary material for: Deletion of afpab1 Causes Increased Sensitivity to Oxidative Stress and Hypovirulence in Aspergillus fumigatus
Source: Int J Mol Sci. 2016 Oct 29;17(11):1811. doi: 10.3390/ijms17111811 (PMC5133812; doi:10.3390/ijms17111811)
Supplement: Supplementary file 1 [file ijms-17-01811-s001.pdf]

Supplementary Materials: Deletion of *afpab1* Causes Increased Sensitivity to Oxidative Stress and Hypovirulence in *Aspergillus fumigatus*

Dongyang Wang, Shunan Wang, Dan He, Song Gao, Baiji Xue and Li Wang

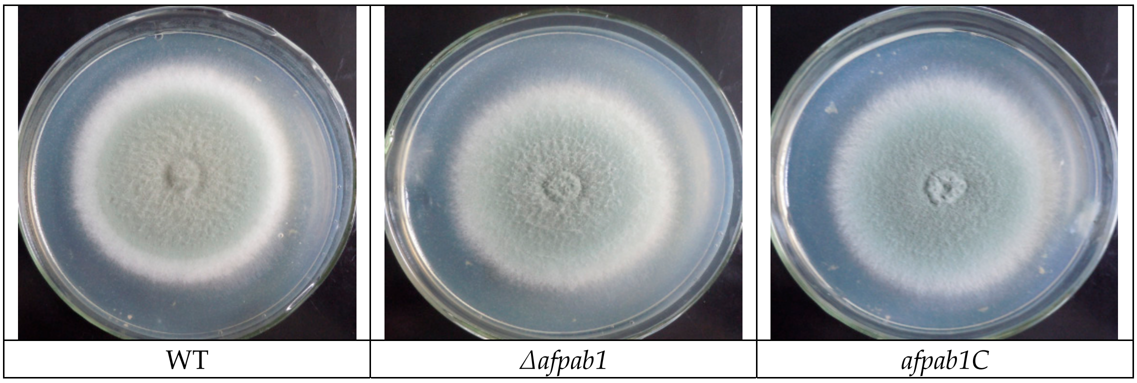

Figure S1. The morphology of colony from WT, *Δafpab1* and *afpab1C* strains on PDA medium at 4 days.

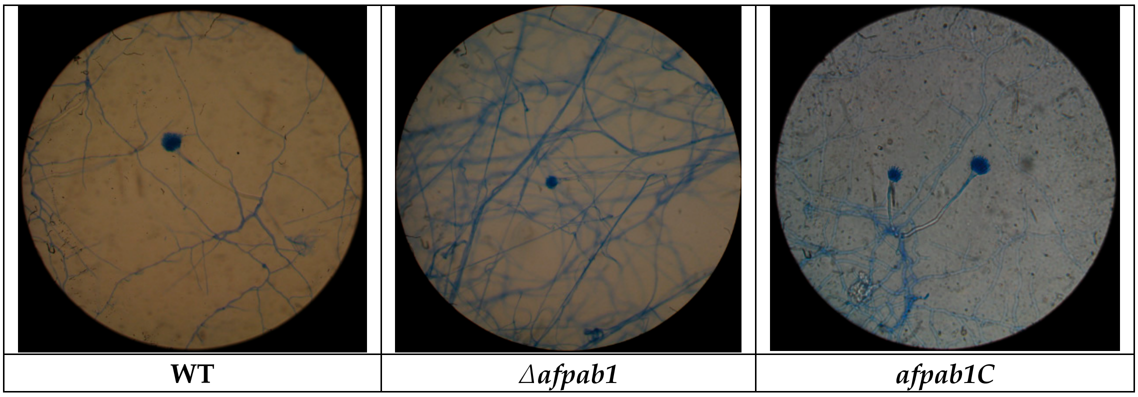

Figure S2. The microscopic morphology from WT, *Δafpab1* and a*fpab1C* strains on PDA medium at 4 days (200×).

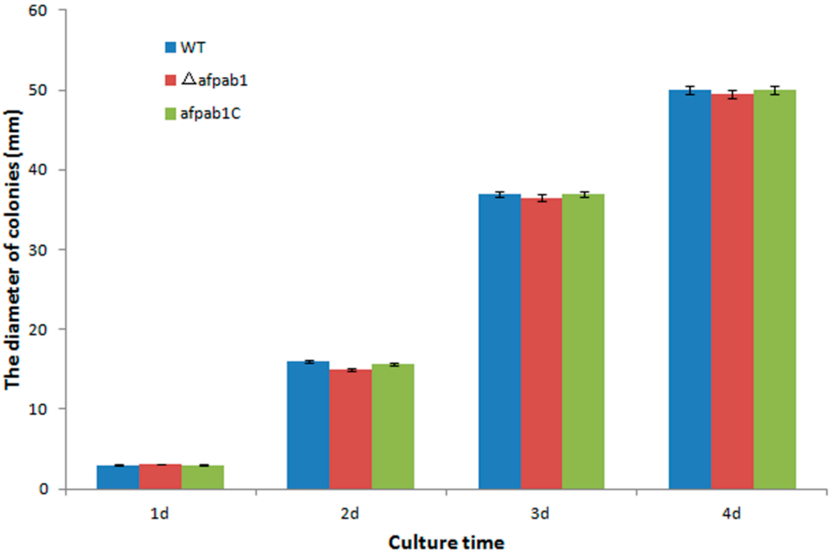

Figure S3. The diameters of colonies from WT, *Δafpab1* and *afpab1C* strains on PDA culture at 28 °C.

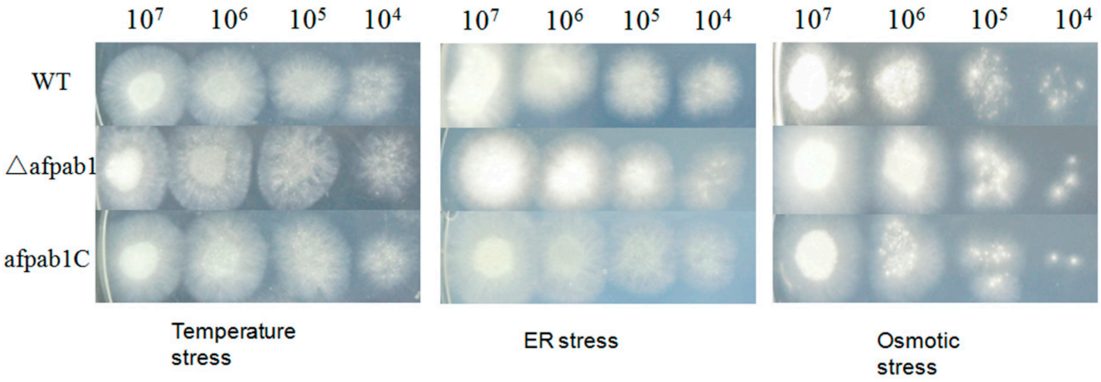

Figure 4. The plate assays from WT, *Δafpab1* and *afpab1C* strains under different stress condition (tempeerature stress, ER stress, Osmotic stress). In general, there is no clear difference under this stress condition between strains.
